# Recurrent individual treatment assignment: a treatment policy approach to account for heterogeneous treatment effects

**DOI:** 10.1038/s41539-021-00117-4

**Published:** 2022-02-04

**Authors:** Ilja Cornelisz, Chris van Klaveren

**Affiliations:** Faculty of Behavioral and Movement Sciences, Amsterdam Center for Learning Analytics, Vrije Universiteit Amsterdam, Amsterdam, The Netherlands

**Keywords:** Economics, Psychology, Decision making

## Abstract

Longitudinal randomized controlled trials generally assign individuals randomly to interventions at baseline and then evaluate how differential average treatment effects evolve over time. This study shows that longitudinal settings could benefit from *Recurrent Individual Treatment Assignment* (*RITA*) instead, particularly in the face of (dynamic) heterogeneous treatment effects. Focusing on the optimization of treatment assignment, rather than on estimating treatment effects, acknowledges the presence of unobserved heterogeneous treatment effects and improves overall intervention response when compared to intervention policies in longitudinal settings based on *Randomized Controlled Trials* (*RCT*s)-derived average treatment effects. This study develops a *RITA-*algorithm and evaluates its performance in a multi-period simulation setting, considering two alternative interventions and varying the extent of unobserved heterogeneity in individual treatment response. The results show that *RITA* learns quickly, and adapts individual assignments effectively. If treatment heterogeneity exists, the inherent focus on both exploit and explore enables *RITA* to outperform a conventional assignment strategy that relies on *RCT*-derived average treatment effects.

## Introduction

Intervention studies examine the effectiveness of a particular intervention relative to the status quo or another competing intervention. Randomization is a crucial element in these studies, as it ensures independence between (un)observed characteristics and the probability of receiving a particular intervention. Due to the randomization, the observed mean outcome differences between the considered interventions can be solely attributed to intervention effectiveness, and this difference is the average treatment effect (ATE). This study shows that the conventional unbiased ATE-estimate can be uninformative for the decision of who should receive which intervention, and provides with the recurrent individual treatment assignment (RITA)-algorithm a viable alternative instead.

The point of departure is that the observed outcome distribution after receiving the intervention (i.e., intervention response) is the result of (1) baseline differences, (2) measurement error, and (3) heterogeneous treatment effects. Heterogeneous treatment effects (HTE) represent the individual variation in intervention response, and can best be described as systematic variability in the direction and magnitude of individual treatment effects (ITEs). There can be considerable individual variation in intervention response when estimating the effectiveness of a particular intervention^1^.

Randomized controlled trials (RCTs) distill the ATE from the intervention response by comparing the outcome means of the different interventions. Although this provides information about which intervention is on average more effective, it does not provide information about which intervention works best for whom. If the educational and clinical practice is personalized then knowing which treatment works best for an individual is critical in making correct intervention assignment decisions^2^.

**Fig. 1:**
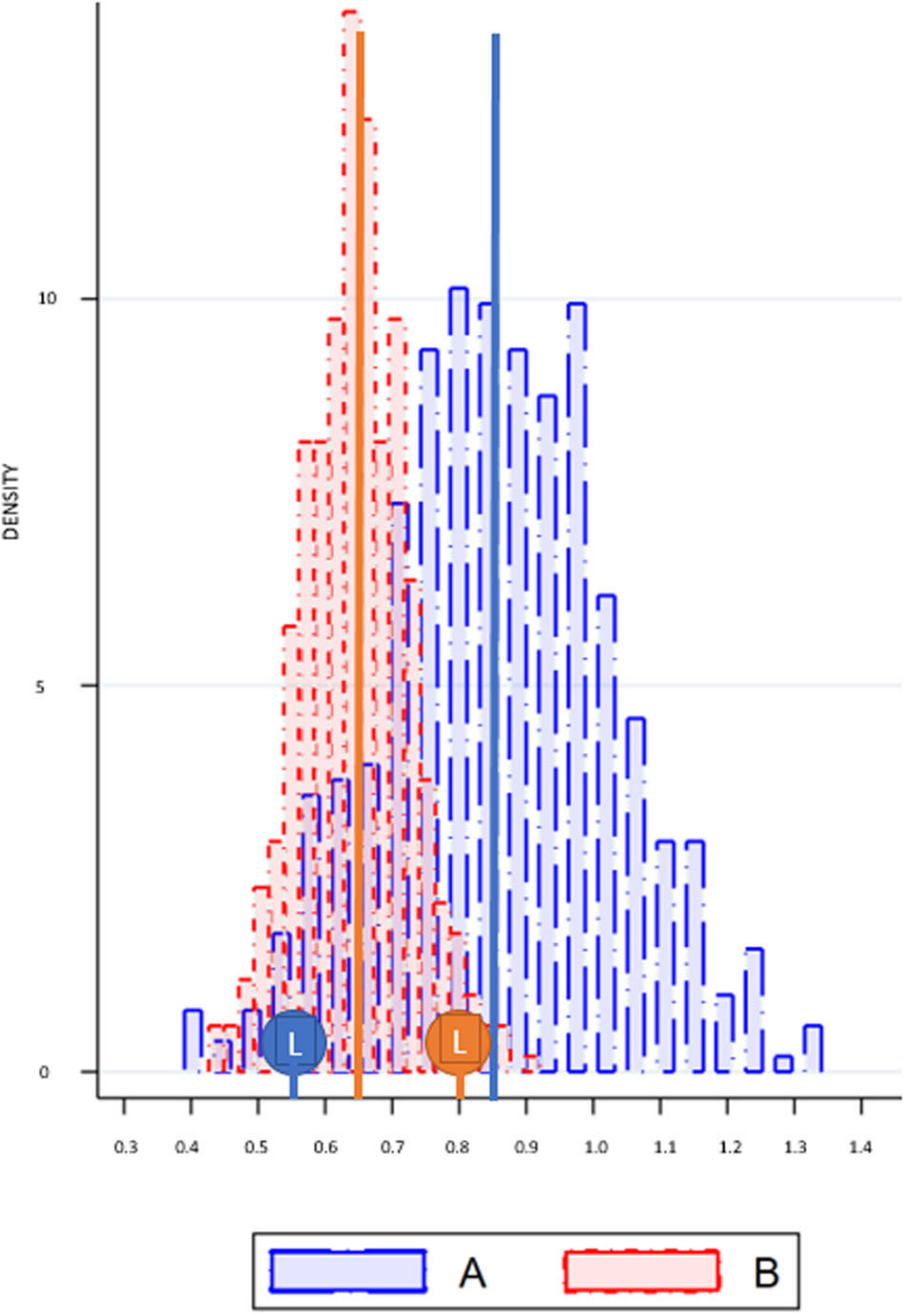
Heterogeneous, average, and individual treatment effects. Hypothetical results of a randomized controlled field-experiment. The outcome distributions show the resulted outcomes after being treated with treatment A (blue) or B (red). The lollypops show the outcomes after being treated with treatment A (blue) or B (red) for one particular person L(ucas).

Using Fig. 1 we intuitively explain the link between *HTE*, *ATE*, and *ITE*, and more importantly how an unbiased *ATE estimate* can be uninformative for the decision whether to assign an individual to intervention *A* or to the competing intervention *B*.

The figure represents the hypothetical results of an RCT conducted to examine the differential effectiveness of interventions *A* and *B*. Let us assume, for expositional purposes, that both interventions represent computerized adaptive practicing algorithms designed to improve children’s reading skills, and that the figure shows the standardized outcomes achieved on a summative test.

Figure 1 reveals that intervention A improves children’s reading skills more than intervention B, with a differential *ATE* of 0.2 standard deviation. A single individual cannot be randomly assigned to both intervention A and B, such that one of both potential outcomes (i.e., *y*_*Ai*_ or *y*_*Bi*_) is observed for each individual which prevents the estimation of the ITE^3^. This is why *RCT*s are frequently considered as the golden standard: randomization followed by a comparison of the generated outcomes yields the (differential) *ATE*. The observed treatment-effect distribution of intervention A can thus be viewed as the counterfactual treatment-effect distribution of intervention B (and vice versa).

For this study, it is important to make a distinction between *ATE* estimation and treatment assignment. Based on the *RCT* results, the conclusion is that intervention *A* is *on average* more effective than intervention B, and this would generally translate into the following treatment assignment rule which states that individuals whose reading outcomes need to be improved can better receive intervention *A* than *B*. The latter conclusion is however tricky given that the figure shows that there is substantial individual variation in treatment response. To illustrate this, Fig. 1 shows the treatment effects of both interventions for Lucas, indicated by the blue and orange lollipops. Even though intervention *A* is on average more effective than *B*, it holds for Lucas that intervention *B* improves his reading outcomes by 0.25 standard deviations more than intervention A. If heterogeneous treatment effects are ignored, it can be dangerous to formulate an individual treatment assignment rule based on an *ATE* estimate, because the *unbiased* *ATE* may provide a *biased* estimate of the *ITE*. Therefore, the mere estimation of the *ATE* is insufficient to determine which intervention should be chosen for an individual in the face of *HTE*^4^. Notwithstanding the overall importance of *ATE*, it may potentially fail to reveal the complex mixture of substantial benefits for some, a little benefit for many, and even harm for a few^5^.

Studies of learning and clinical studies have less formally also brought this point forward. It has, for example, been pointed out that there are infinite ways to personalize (computerized adaptive) learning and optimal adaptation of the content offered to a heterogeneous group of learners cannot be realized when considering only a single algorithm or by evaluating only the *ATE*^6^. Furthermore, it has been emphasized that taking into account heterogeneous treatment effects in adaptive practice environments is crucial for the development of effective personalized practicing environments and the understanding of the underlying learning mechanisms of these environments^7^.

Other studies mentioned that challenges caused by *HTE* (e.g., reference class problem, statistical over-fitting, false discovery, biased predictions for new populations) must be solved first before estimated *ATE*’s can be used to guide individual clinical and educational decision-making^1,8^.

At the same time, the results of observational studies without randomization do not provide a proper alternative because estimation parameters based on these data might not be internally valid and will thus not result in improved treatment assignment rules^9^. Relative to deciding on one-off interventions, multi-period—or longitudinal—*RCT*-settings have the appealing feature that *learning over time* is possible, which offers opportunities to deal with heterogeneous treatment effects^10^.

Learning over time can happen through *exploitation* and *exploration*. Learning by exploitation implies that previous observations are used to make better treatment assignment decisions. Learning through exploration implies that individuals are randomly assigned to different interventions to infer what intervention (still) works best for whom. Choosing between these two types of learning inherently imposes an explore-exploit trade-off and machine learning (ML) and Bayesian techniques are increasingly used to make such data-driven treatment assignment decisions, with a rapidly growing body of literature investigating so-called ‘bandits’^11^.

These algorithms learn over time which intervention is relatively the most effective with which certainty and more certainty about the relative effectiveness implies that the algorithm will explore less and exploit more. These models will always explore to a certain extent to account for dynamic treatment effects (i.e. changing relative effectiveness of the interventions over time). Even though this is not the topic of this study, we note that also the *RITA* algorithm proposed always explores to account for dynamic treatment effects.

Currently, existing algorithmic approaches focus on the estimation of the individual treatment effect (i.e., a deterministic or frequentist approach) or on determining the optimal individual treatment assignment probability (i.e., a stochastic, or Bayesian/bandit approach). The frequentist approach correctly assumes that ITEs are pre-determined but unobserved and potentially heterogeneous. To account for potential heterogeneity, moderator effects are included in the estimation model and the frequentist approach then (1) determines for each considered subgroup the intervention that is relatively most effective, and (2) randomly assigned persons to this intervention with, for example, probability 0.95 as to learn in the feature if this intervention remains relatively most effective. Bandit approaches incorrectly assume that there is a probability that one intervention is relatively most effective, but by doing so, this approach ensures that future treatment assignments are conditionally determined on background features. It holds that the explore-exploit trade-off and taking into account heterogeneous treatment effects are intertwined in this stochastic approach. Whereas much progress has been made in the ‘Bandits’ literature, still there is much to learn about optimal individual treatment assignment when individuals have specific (unobserved) characteristics and are observed over time^10^.

There are two key challenges for both the frequentist and stochastic approaches. First, both approaches account for *HTE* by conditioning only on available observed characteristics, which raises bais concerns in that unobserved characteristics might influence both intervention status and outcome observations. While particularly the algorithmic advances in estimating individual treatment assignment are promising, both approaches rely on stark assumptions, such as *strong ignorability* or *no unmeasured confounding*^12,13^.

Second, when these methods condition on a large set of background characteristics to account for HTE, the conditioning may suffer from generalization concerns as a result of unobserved characteristics which can invalidate the apparent equivalence (i.e., based on observable characteristics) of individuals. While recent methodological approaches using rich data can increasingly distinguish between different sub-populations, this so-called reference class problem remains largely unresolved and merits additional research^1^.

The current state of affairs is therefore that current approaches used to account for *HTE* suffer from both internal (bias) and external (reference class) problems.

The proposed algorithm in this study acknowledges that the ultimate objective of longitudinal individual treatment assignment is not to estimate conditional *ITE*-proxies (which in reality represent subgroup *ATE*s) or conditional assignment probabilities (which in reality represent subgroup assignment probabilities) as this directly prevents the possibility to take into account unobserved heterogeneous treatment effects. Instead, the intuition is that recurrent assignment decisions should be based on learning—over time—what the optimal intervention is for each individual. If individual treatment assignments improve longitudinally, then this will be reflected by improvements in individual-level and population-level treatment response, without necessitating *ITE* inference-making. The ‘Recurrent Individual Treatment Assignment’ (*RITA*)-algorithm is a longitudinal individual assignment algorithm based on sequential *RCTs* or A/B-tests and observed variation in intervention response. *RITA* updates assignment decisions based on random variation in treatment assignment for a specific individual as well as others, and treatment response variation across alternative interventions. By focusing on treatment response variation, RITA can avoid reference class issues and accommodate unobserved heterogeneity in treatment effects.

This study illustrates RITA and the different stages of decision rules associated with it. Simulation results for *RITA* are presented that focus on a multi-period setting (60 periods) and in which heterogeneous treatment effects are considered for two alternative interventions (*A* and *B*) in a population of 1000 individuals. Throughout the paper, and without loss of generality, one can think of these two alternative interventions as educational interventions (e.g., two competing computerized adaptive practicing programs) or as clinical interventions (e.g., two alternative cancer medicines). Four different outcome worlds are considered that differ in the extent of heterogeneity in treatment effects, whether this heterogeneity is (partially) unobserved, and in the relative treatment effects of both interventions. The performance of *RITA* is evaluated and compared against the baseline model in which individuals are assigned to the intervention based on an *RCT*-derived ATE instead. The simulation results indicate that *RITA* has the potential to learn over time what the best individual treatment assignment decision is. When confronted with heterogeneous treatment effects, this approach will improve overall treatment response, relative to a baseline approach based on *RCT*-derived ATEs. Intuitively, better performance of *RITA* is the net result of an asymmetry in that the benefits for individuals who benefit from sequential exploration tend to be much larger than the losses for individuals who do not.

The RITA algorithm can be implemented in high-iterative educational and clinical settings. Online or digital educational environments, for example, lend themselves exceptionally well for addressing heterogeneity with a RITA algorithm. Practicing environments (e.g., Math Garden, Newton, Duolingo) currently return exercises to students using different rules/algorithms, and RITA could be used within these environments to test which adaptive rule/algorithm works best for whom. Similarly, RITA can also be used to learn which feedback or nudges work best for whom and, subsequently and simultaneously, it can assign the type of feedback and nudges to students that are most effective for them. Machine Learning and Bandit-algorithms are more and more implemented in clinical and educational practice, and it generally holds that RITA can be implemented in these settings as well, as it can better handle the heterogeneity and as such can more effectively assign persons to the treatment that is most effective for them.

Section “Simulation data” describes the four different heterogeneous environments that are simulated. Section “Simulation results” compares the relative performance of RITA to the baseline model (i.e., treatment assignment using a randomized controlled trial) for the four different simulated environments. Section “Comparing *RITA* with more advanced models” outlines how the relative performance changes if the relative performance of RITA is compared to more advanced existing algorithms that can address observed heterogeneity. Section “Discussion” concludes and discusses potential implications and future research avenues. Finally, Section “Methods” gives a technical description of the Randomized Controlled Trial baseline model and the RITA algorithm.

## Results

### Simulation data

To illustrate the relevance of applying *RITA* in the face of *HTE*, four different treatment effect settings (referred to as “worlds”) are simulated for which the performance of *RITA*—relative to the baseline *RCT* algorithm—is evaluated in terms of treatment assignment, cumulative treatment response, and individual treatment response. The intuition is that, without being informed about the specific heterogeneous character of both treatment effect distributions, the objective of the baseline model and *RITA* is to improve outcomes by assigning individuals to either intervention *A* or *B*. In the section “Comparing RITA with more advanced models” a formal representation of the *RITA* algorithm is provided.

**Fig. 2:**
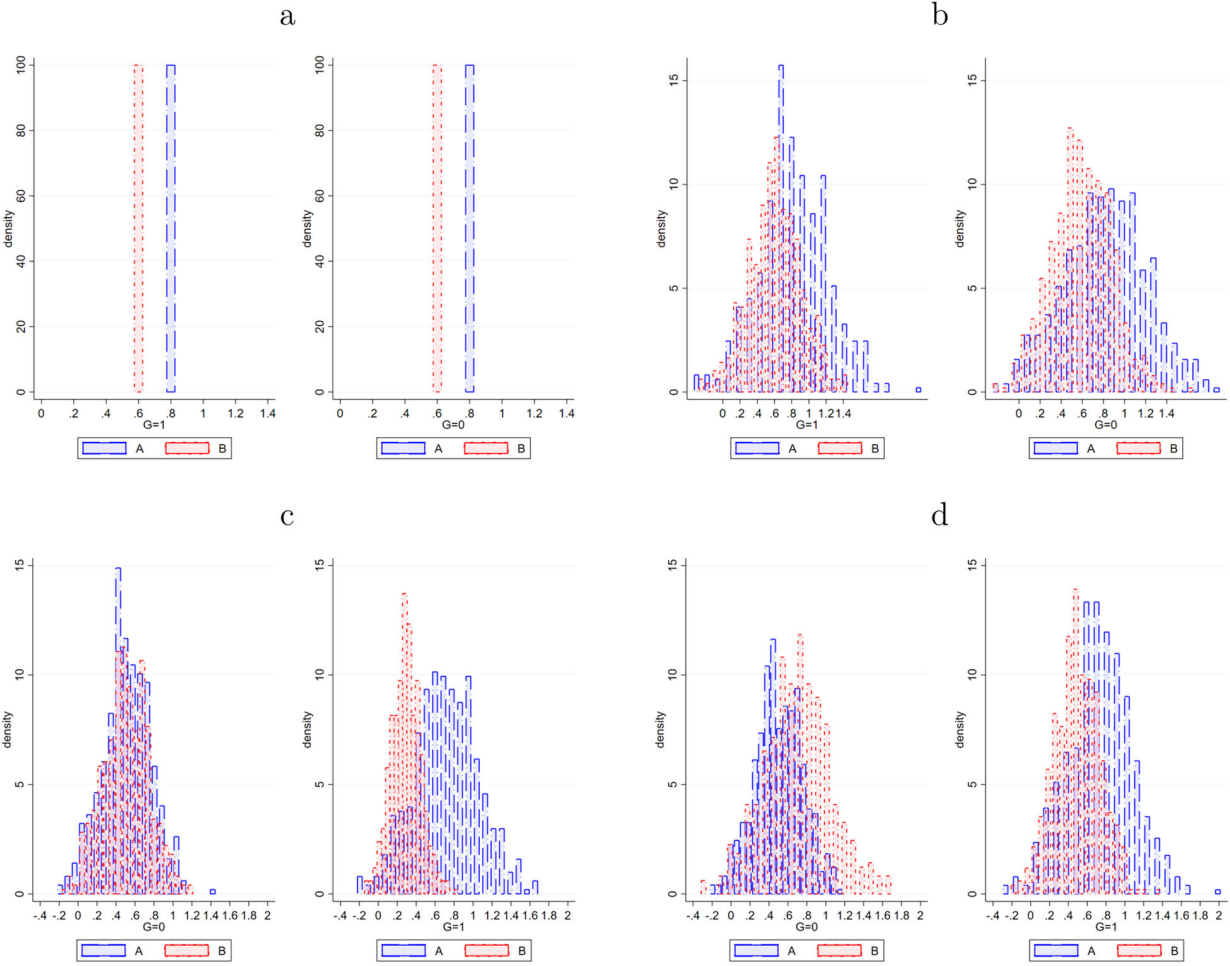
Simulated distributions of treatment effects. **a** World 1: Constant treatment effects with mean treatment effect of A = 0.8 and B = 0.6. **b** World 2: Treatment effects with mean treatment effect of A = 0.8 (and heterogeneity *e*_*LAi*_  ∼ *N*_*A*_(0.8, 0.4)) and B = 0.6 (and heterogeneity *e*_*LAi*_  ∼ *N*_*B*_(0.6, 0.3)). **c** World 3: Treatment effects with mean treatment effect of A = 0.8 (and heterogeneity $e_{LAi}\,{\sim}\,{N_A} {(0.7, 0.35)} +\frac{e_{LAi}\cdot G}{3.5}$) and B = 0.6 (and heterogeneity $e_{LBi}\,{\sim}\,{N_{B}} {(0.6, 0.3)} +\frac{e_{LBi}\cdot (1{-}G)}{2.5}$). **d** World 4: Treatment effects with mean treatment effect of A = 0.8 (and heterogeneity $e_{LAi}\,{\sim}\,{N_{A}} {(0.7, 0.35)}+ \frac{e_{LAi}\cdot G}{3.5}$) and B = 0.8 (and heterogeneity $e_{LBi}\,{\sim}\,{N_{B}} {(0.7, 0.35)}+ \frac{e_{LBi}\cdot (1{-}G)}{3.5}$).

Table 1 indicates how (heterogeneous) treatment effects for interventions *A* and *B* are generated in each of the four worlds. The third column points out the mean treatment effects and columns four and five describe the nature of the heterogeneity. For convenience, and without loss of generality, we impose $$\begin{array}{c}{h}_{I}({L}_{i},{X}_{i})={u}_{I}({L}_{i})+{u}_{I}({L}_{i})\cdot {o}_{I}({X}_{i})\end{array}$$ as the structure of heterogeneity, such that the simulated heterogeneous treatment effects can be described by an unobserved (*L*atent) and an observed component related to *X*. In Fig. 2, the resulting treatment effect distributions are presented. Naturally, both algorithms do not observe these different treatment-effect distributions when completing their task of determining optimal treatment assignment (since assignment would be trivial if *ITE* for both treatments is known and observed by the treatment assignment algorithm).

**Table 1:** Simulation worlds.

World	Treatment	Mean effect	Heterogeneity	
			*h*_*Ii*_(*L*_*i*_, *X*_*i*_) = *u*_*Ii*_(*L*_*i*_)+*u*_*Ii*_(*L*_*i*_) ⋅ *o*_*Ii*_(*X*_*i*_)	
			*u*_*Ii*_(*L*_*i*_)	*u*_*Ii*_(*L*_*i*_) ⋅ *o*_*Ii*_(*X*_*i*_)
1	*A*	0.8	0	0
	*B*	0.6	0	0
2	*A*	0.8	*e*_*LAi*_ ~ *N*_*A*_(0.8, 0.4)	0
	*B*	0.6	*e*_*LBi*_ ~ *N*_*B*_(0.6, 0.3)	0
3	*A*	0.8	*e*_*LAi*_ ~ *N*_*A*_(0.7, 0.35)	${u}_{Ai}({L}_{i})\cdot \frac{G}{3.5}$
	*B*	0.6	*e*_*LBi*_ ~ *N*_*B*_(0.5, 0.25)	${u}_{Bi}({L}_{i})\cdot \frac{1-G}{2.5}$
4	*A*	0.8	*e*_*LAi*_ ~ *N*_*A*_(0.7, 0.35)	${u}_{Ai}({L}_{i})\cdot \frac{G}{3.5}$
	*B*	0.8	*e*_*LBi*_ ~ *N*_*B*_(0.7, 0.35)	${u}_{Bi}({L}_{i})\cdot \frac{1-G}{3.5}$

World 1 represents a world without heterogeneous treatment effects and for all individuals, intervention *A* is more effective than intervention *B* by a difference of 0.2 in treatment response in each period. World 2 represents a situation in which ATE for both treatments is the same as in World 1, but in World 2 there is unobserved heterogeneity in treatment effects. This heterogeneity is normally distributed around the mean treatment effect with a standard deviation that is half the *ATE* (i.e., a coefficient of variance of 0.5). World 3 represents a situation with both unobserved and observed heterogeneity, in which both dimensions interact through a grouping variable *G* (*G* = 0, 1) that is equally distributed across the population. As such, for the group *G* = 1, the average effect for intervention *A* is ${h}_{A,G = 1}({\overline {L}},1)=0.7+0.7* \frac{1}{3.5}=0.9$, for *G* = 0, the ATE is ${h}_{A,G = 0}({\overline {L}},0)=0.7+0.7* \frac{0}{3.5}=0.7$, yielding again an overall *ATE* of 0.8. Similarly, intervention *B* has an ATE of 0.5 if *G* = 1 and 0.7 if *G* = 0, yielding again an overall *ATE* of 0.6. World 4 is a special context in which interventions *A* and *B* have the same *ATE* (i.e., 0.8), but intervention *A* (*B*) is on average more effective for group 1 (0). World 1 and 4 are in a way each other’s counterparts; while obtaining information about the *ATE* is critical (and sufficient) for effectively assigning individuals to the most effective treatment in World 1, this information is of no value in World 4 as a result of *ATE* equivalence for intervention *A* and *B*.

Figure 2 illustrates the treatment assignment challenge explained in the introduction in that—if heterogeneity is present— intervention *B* can be better for an individual even if intervention *A* has a higher *ATE*. The quest for both treatment assignment algorithms is to discover how individuals can be effectively assigned to the most effective intervention. The only information given to both models in period 1 is (1) baseline outcome scores at *t* = 0, (2) an initial random treatment assignment, and (3) treatment response in each period conditional on treatment assignment. These initializing values were ${P}_{i}(A)={P}_{i}(B)=\frac{1}{2}$ and *y*_0*i*_ ~ *N*(10, 1). In each period, observed variation in treatment response is then the result of unobserved variation in treatment effectiveness and other unobserved factors, with the latter (error term) represented by a draw from a normal distribution with mean zero and standard deviation of 0.1 (*ϵ*_*ti*_ ~ *N*(0, 0.1)). Furthermore, 60 periods are considered for evaluating the performance of both algorithms. This is—of course—arbitrary, but could for example be thought of as a period of 5 years with monthly updates regarding treatment response.

Given that heterogeneity is absent in World 1, present in World 2 and 3 but with a non-zero differential *ATE*, and that there is *ATE* equivalence in heterogeneous World 4, it is expected that the relative benefit of applying *RITA* will improve moving from World 1–4. Instead, the opposite is true for the baseline model which will perform best in a world absent of heterogeneity (i.e., *ATE* applies to all individuals).

### Simulation results

**Fig. 3:**
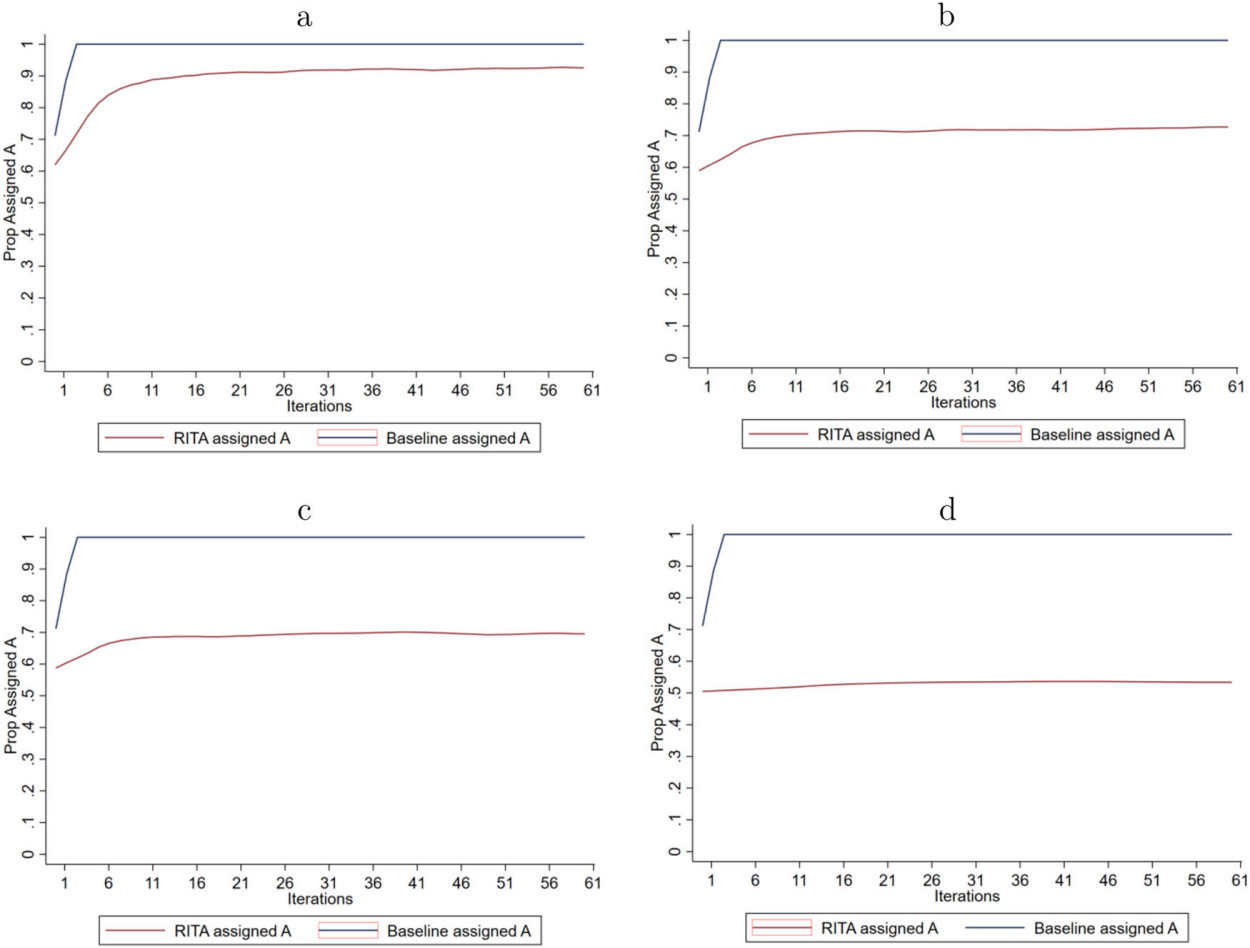
Longitudinal treatment assignment. **a** Results for World 1. **b** Results for World 2. **c** Results for World 3. **d** Results for World 4.

The objective of the baseline model is to first obtain an unbiased estimator of the *ATE* and determine treatment assignment accordingly, while the objective of *RITA* is to assign individuals recurrently to the most effective intervention without explicitly modeling treatment effects. Figure 3 shows the resulting differences in treatment assignment between these two approaches for all four Worlds. The vertical axis represents the proportion assigned to treatment *A* and the horizontal axis refers to the number of periods.

The baseline model learns in the first period for Worlds 1–3 that intervention *A* is on average significantly more effective than intervention *B*. Based on this information, individuals are assigned to intervention *A* with certainty (i.e., a probability of 1) for the remaining periods. Figure 3, therefore, shows for the baseline model that all persons receive intervention *A*. In World 4, the ATE of both interventions is the same and the assignment rule applied then is to assign individuals to the intervention that is coefficient-wise most effective, regardless of statistical (in-)significance. The intuition is that due to the insignificance in differential treatment effectiveness it is irrelevant which intervention is assigned, but the baseline algorithm must formulate a specific assignment rule based on the size of the estimated treatment effect coefficient (i.e., *α* in Eq. (3)). Treatment assignment by *RITA* differs from the baseline model and Fig. 3 shows that this distinction is increasing in the presence of *HTE* and in the absence of a differential *ATE* between intervention *A* and *B*. Figure 3 clearly indicates that *RITA* assigns individuals differently, which is necessary but insufficient for improving outcomes. As such, it does not reveal whether RITA is actually more effective than a baseline model approach.

**Fig. 4:**
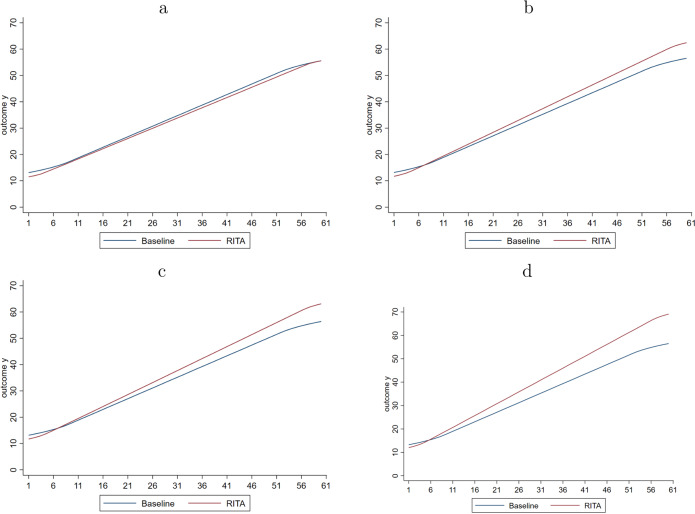
Average cumulative outcomes. **a** Results for World 1. **b** Results for World 2. **c** Results for World 3. **d** Results for World 4.

Figure 4 visualizes the average cumulative outcome gains over periods for both assignment strategies. These results indicate that *RITA* performs better than the baseline model in all worlds, except in World 1. The relatively better performance of the baseline model in World a has two apparent reasons. First of all, there is no heterogeneity in World 1, such that intervention *A* is the most effective intervention for *all* individuals. The baseline model immediately reacts optimally to this situation in the first period by concluding that all individuals ought to be assigned intervention *A* subsequently in a deterministic fashion. Instead, whereas *RITA* updates assignment probabilities considerably in favor of intervention *A*, it does not immediately converge to a fully-deterministic assignment probability of 1 for everyone after only one period. Secondly, *RITA* explicitly trades off the objective to offer individuals the most effective intervention at time *t* (*exploit*), and take into account the possibility of individual dynamic treatment effects (*explore*), as indicated by Step 6 of the algorithm. While the simulations presented here focus on heterogeneous treatment effects in the absence of dynamic treatment effects, *RITA* allows for future dynamics in *ITE*. Maintaining such exploration comes at the expense of model performance if no such dynamics occur.

Worlds 2 and 3 highlight that *RITA* uses the first handful of periods to update treatment assignment probabilities, by taking into account the unobserved and observed heterogeneous treatment effects. After approximately 7-8 periods, average cumulative gains by *RITA* are similar to the baseline model approach, after which results start to diverge in the advantage of *RITA*. This is the result of acknowledging variation in relative treatment effectiveness across individuals. In World 4, when only observing *ATE* is no longer informative for individual treatment assignment, the relative performance of *RITA* is even better. The increase in cumulative gains in World 4 by applying *RITA* -instead of the baseline model approach- is on average 11.45 (SD = 21.08). Whereas average cumulative output gains are important, it does not highlight whether individuals necessarily benefit from *RITA* when heterogeneity of treatment effects is present.

**Fig. 5:**
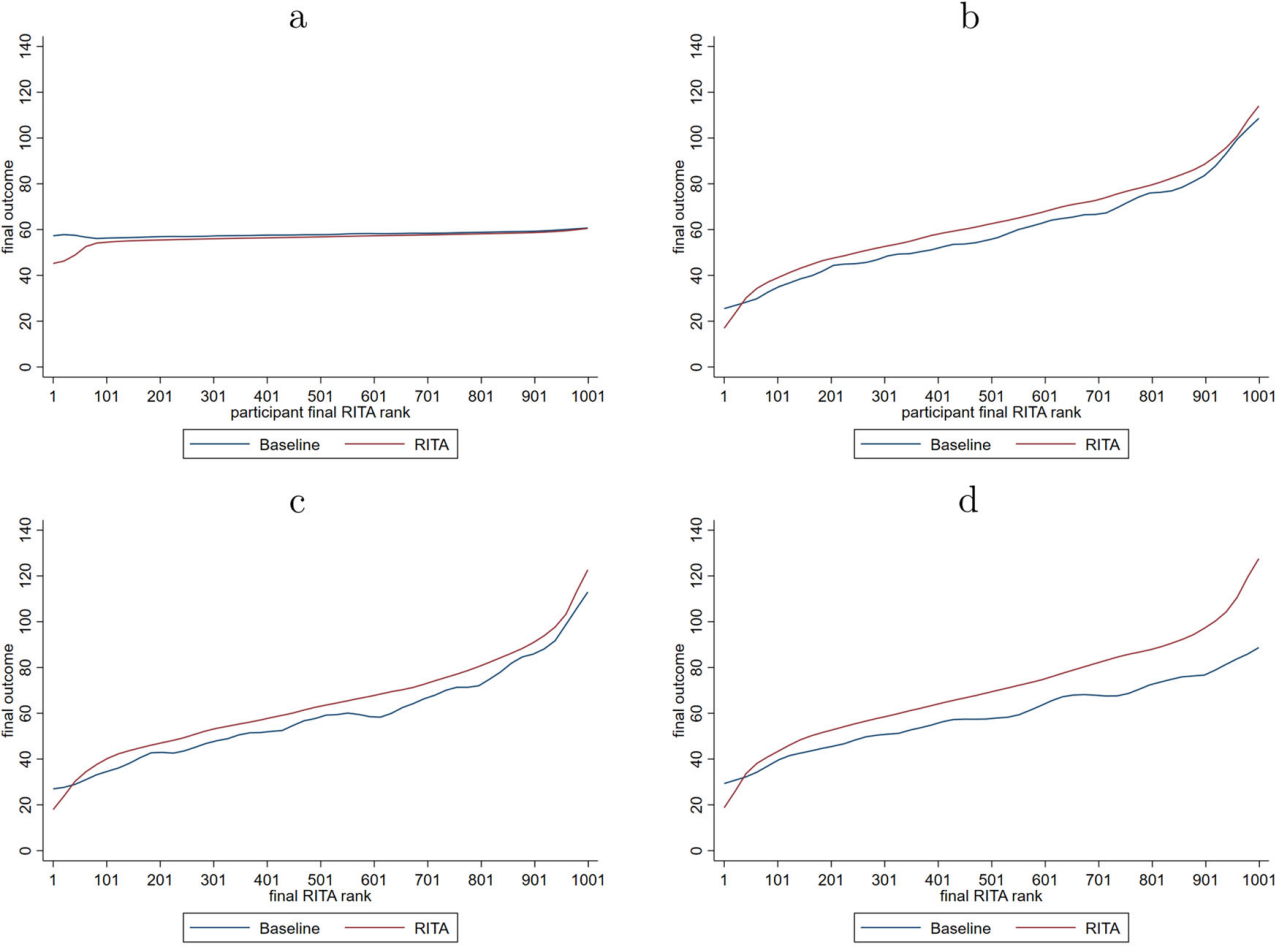
Individual cumulative outcomes. **a** Results for World 1. **b** Results for World 2. **c** Results for World 3. **d** Results for World 4.

Figure 5 shows the individual cumulative outcome gains for each world in which individuals are ordered by their so-called *"RITA* rank”, which is 1 (1000) for the individual with the lowest (highest) final outcome when *RITA* is applied. In Worlds 2–4, applying *RITA* results in better individual cumulative outcomes across a large range of these *RITA-*ranked individuals. Only among the lowest rank bins are individuals considerably worse off when applying *RITA*. These are individuals that experience a relatively unlucky draw of sequential treatment assignments through *RITA* and would benefit from the deterministic assignment to intervention *A* occurring in the baseline model. This drawback of *RITA* is naturally most pronounced in World 1, in which no heterogeneity is present and sequential exploration does not yield benefits. As a result, overall outcomes are generally small, with a median loss of −1.00, indicating that the typical individual has been assigned the wrong intervention—here intervention *B*—5 times in the total sequence of 60 periods. From the treatment assignment proportions displayed in Fig. 3, it can be inferred that such wrong assignments particularly occur in the first 7–8 periods as a result of updating assignment probabilities and later on as a result of continuous exploration (i.e., RCTs).

In fact, individuals for whom intervention *A* is optimal generally lose somewhat as a result of this explorative nature of recurrent treatment assignment and this is observed across all Worlds. But, these—generally small—losses are more than offset by the relatively large benefits observed for similarly ranked individuals for whom intervention *B* is better. Furthermore, *RITA* particularly performs well when the two interventions are evenly effective on average, but with (large) unobserved differences in individual relative treatment effectiveness (i.e., World 4). In this World 4, individuals (*n* = 520) for whom intervention *A* is better to experience a small loss when *RITA* is applied, with a median loss of −0.71 over 60 periods. In contrast, individuals (*n* = 480) for whom intervention *B* is better to experience a potentially huge benefit when *RITA* is applied, with a median gain of 21.91 over 60 periods. In sum, the results point to an important asymmetry in that small loss for individuals who do not benefit from exploration are more than offset by large gains for individuals who do benefit from such iterative updates in treatment assignment.

### Comparing *RITA* with more advanced models

In the study at hand, we compared RITA with a simple baseline model. The simulation results show that the baseline model outperforms RITA in the absence of *HTE* and with one intervention being more effective than the other and that RITA outperforms the baseline model when treatment effects are heterogeneous. We could also have compared *RITA* with more advanced algorithms that can address observed heterogeneity. The two comparison models that appear to be most intuitive to use would be (1) a baseline model with an interaction-effect included (i.e., a frequentist approach), and (2) a Bandit/Bayesian model in which the assignment probability is updated conditionally on the observed heterogeneity (i.e., *o*_*Ii*_(*X*_*i*_)).

In this section, we argue that the simulation results are shown in the previous section already indicate how the performance of these models would relate to the performance of the baseline model and RITA. Let us assume that there is only observed heterogeneity which is captured fully by ***x***. The baseline model with an interaction term would pick this observed heterogeneity up in the first period and would assign all persons to the correct treatment after period 1. The Bayesian or bandit approaches will figure out the optimal treatment assignment as well but will converge slower to the optimal assignment strategy. Also, RITA will figure out the optimal treatment assignment but not as effective as the baseline model with the interaction term included. The more complex the observed heterogeneity will be the more Bayesian and RITA models will have the advantage. It is more realistic to assume that much of the heterogeneity is unobserved. If we would assume that there is only unobserved heterogeneity, then no interaction can be added, and no treatment assignment probability can be updated conditionally on an observable. It implies that RITA outperforms all other models as it is the only model that can handle unobserved heterogeneity. In the simulations, the actual outcome is not so important, because these outcomes are substantially driven by the outcome variables chosen, the extent of observed and unobserved heterogeneity present, and specific settings and choices concerning the tuning of the hyperparameters of the models (e.g., form of the learning parameter, the modeling of the Bayesian updating process, the number of interaction terms allowed, etc.). The key insight of our model is that by using a rank model we can account for unobserved and observed heterogeneity, while currently, existing models can account only for observed heterogeneity. To increase the readability of the study at hand, and without loss of generality, we, therefore, showed the results of the most ‘extreme comparisons’. In many educational and clinical settings, the results of one-shot RCTs are used for future treatment assignments. This study shows that taking into account observed and unobserved heterogeneity would improve treatment assignment and thereby increase intervention effectiveness.

## Discussion

The importance of RCTs in generating unbiased *ATE*s, as highlighted by Rubin’s potential outcome model, has been widely acknowledged and dominant in developing the research field of evaluating interventions and evaluation guidelines^1,3,14^. Yet, when there is considerable individual variation in intervention response when estimating the effectiveness of a particular intervention (i.e., heterogeneous treatment effects) using ATE results can misguide the attempts at effectively personalizing intervention; yielding potentially substantial benefit for some individuals, a little benefit for many, and even harm for a few^4,5^. Given that the ATE can be non-informative for individual treatment assignment, learning what the individualized treatment rules ought to be instead is methodologically challenging in that it requires causal results *and* accurate prediction estimations regarding ITE^9^. It holds that ITEs are fundamentally difficult to determine; even when rich longitudinal information is available.

This study departs from acknowledging that the ultimate objective of longitudinal individual treatment assignment is *not* to try and estimate the individual effect of interventions explicitly but to apply a decision rule that is based on variation in treatment assignment and treatment response over time. We combine insights from predictive and causal explanatory modeling and develop the *RITA* algorithm, which performs sequential *RCT*s in which treatment assignment probabilities are updated after each iteration. These updates are based on variation in treatment response, such that it captures both observed and unobserved treatment effect heterogeneity. The intuition is that recurrent assignment decisions should be based on learning—over time—what the optimal treatment assignment is for each individual, without necessitating attempts at modeling ITE explicitly. An approach based on *RITA* trades off *exploit* (i.e., updating treatment assignment probability over time) and *explore* (i.e., RCT-evaluations) to both benefit from what has been learned so far and to continue to learn from future treatment response observations.

To evaluate the potential of *RITA*, this study introduced a particular example of such an algorithm, and compared performance to a baseline model in which individuals are assigned to treatment on the basis of an *RCT*-derived ATE instead. A total of four different settings are simulated that differ importantly in the extent of heterogeneity in treatment effects, whether this heterogeneity is (partially) unobserved, and in the relative treatment effectiveness of both treatments. The simulation results show that the treatment assignment decisions of both algorithms differ substantially and that *RITA* can learn rapidly what the best treatment assignment is.

Importantly, *RITA*’s overall assignment decisions and relative performance depend strongly on the presence of *unobserved* heterogeneous treatment effects. In the absence of heterogeneity, such an approach may cause individuals some harm due to its sequential exploration, whereas deterministic assignment to the more effective intervention would have been optimal for everyone. Even in the presence of heterogeneity, *RITA* will still relatively harm those individuals that would have been better of with deterministic assignment to one single intervention. Yet, the results point to an important asymmetry in that small loss for these individuals who do not benefit from probabilistic exploration is more than offset by large gains for individuals who do benefit from such iterative updates in treatment assignment probability.

The simulation results thus showed that *RITA* can outperform the more conventional assignment approach based on *RCT*-derived ATEs, by taking into account unobserved treatment effect heterogeneity. This is of particular importance when acknowledging that—in reality—such heterogeneity is generally considerable and its characteristics generally unknown^1^. The implication of this is that, while it can be possible to afterward identify individuals that would have benefited from a different (deterministic) policy, this is generally not possible beforehand. The results thus reveal that many individuals could benefit from RITAs based on probabilistic assignment, as opposed to the conventional assignment approach.

Despite the promising results presented here, many questions are still unresolved. One specific *RITA* algorithm has been presented here and this—by no means—implies that this is the desired or optimal algorithm. Questions regarding asymptotic properties, convergence, and optimality remain still to be investigated. Furthermore, the contexts evaluated here are based on arbitrary parameter settings. It would be crucial to see how this approach performs in settings derived from real-life examples.

Another important behavioral feature of RITA is that persons can be assigned back and forth between the two interventions if rankings are approximately similar (i.e., oscillating behavior). It depends on the specific context, whether this oscillating behavior is problematic. For example, in online educational adaptive practicing software, students might switch between receiving exercises drawn from different “skill sets”, which is not at all problematic for the adaptive system to perform and might also not be problematic for the student, as the intervention assigned are never visible for the student. Also in clinical practice, (with the assignment of medicine) oscillating behavior is not per se problematic, as doctors often have the opportunity to update each month which medicine should be used. Currently, patients who use medicine have their dose box, in which the medication is dosed based on the doctor’s advice. However, if the (mental) costs of switching become higher then oscillating behavior may be problematic. For example, if children are assigned to different learning methods, and RITA assigns some students back and forth between two very different learning methods each day, then this is an undesirable characteristic, because it puts an unnecessary demand on the pupil and the school. A solution within the RITA environment would be to built-in a caliper (i.e., a ranking space is defined such that switches can occur only if the ranking differs by more than, for example, a certain number), such that undesirable oscillating behavior is mitigated.

This study might also inspire future research avenues. Whereas—in this paper—treatment effect heterogeneity has been assumed constant within individuals over time, *RITA’s* inherent feature of continuous exploration can imply this is also a useful approach for determining a dynamic treatment regime in which the level and type of treatment can be tailored to an individual’s changing status over time^12^. Lastly, while the results presented here have focused on improving individual treatment assignment, optimizing treatment assignment over time can reveal underlying treatment effect mechanisms at the level of the individual. This begets the question to investigate the potential for a *RITA* algorithm to support the diagnosis^15^.

## Methods

### Baseline model

Suppose that a *RCT* is conducted to estimate the differential effectiveness of the interventions *A* and *B* on outcome *y*_*i*_ for individual *i* (with *i* = 1, ⋯ , *N*). Let intervention indicator *I* be 1 if individual *i* received intervention *A* and 0 otherwise, and let higher values for *y*_*i*_ represent a more favorable treatment response. The two potential outcomes for individual *i* can then be represented by

1 $${y}_{i}=\left\{\begin{array}{ll}{y}_{Ai}\quad &{\mathrm {if}}\ {I}_{Ai}=1\\ {y}_{Bi}\quad &{\mathrm {if}}\ {I}_{Ai}=0\end{array}\right.,$$

and the individual differential treatment effect as

2 $${y}_{Ai}+({y}_{Bi}-{y}_{Ai})\cdot {I}_{Ai}.$$

The *individual* treatment effect (ITE) can—however—not be assessed, as for each individual *i* only one of both potential outcomes *y*_*Ai*_ or *y*_*Bi*_ is observed.^3^ Therefore, individuals are randomly assigned to one of both intervention, such that the *average* differential treatment effect can be empirically estimated by means of ordinary least-squares regression:

3 $${y}_{i}=\alpha {I}_{Ai}+{{{{\boldsymbol{X}}}}}_{{{{\boldsymbol{i}}}}}^{\prime}{{{\boldsymbol{\beta }}}}+{\varepsilon }_{i}.$$

The parameters *α* and *β* are to be estimated, *ε* represents classical measurement error, which is assumed to be identically and independently distributed with mean 0 (i.e., *ε*_*i*_ ~ iid(0, *σ*^2^)), and matrix ***X*** represents a constant term and represent background characteristics that are included to gain model precision. In Equation (3), the parameter of interest is *α* and if this parameter estimate is—for example—significant and positive, the results indicate that intervention *A* is *on average* more effective than intervention *B*.

To examine whether a differential effect between *A* and *B* persists—or varies—over time, a longitudinal test can be conducted that involves repeated follow-up observations. In these repeated measures estimation models, intervention status is generally time-invariant, with outcome *y* measured repeatedly over time. More formally, such a repeated measures estimation model can be represented by the following Eqs. (4) and (5):

4 $$\left(\begin{array}{l}{y}_{1i}\\ {y}_{2i}\\ \vdots \\ {y}_{Ti}\end{array}\right)=\left(\begin{array}{llll}{I}_{Ai}&0&\ldots &0\\ 0&{I}_{Ai}&\cdots \ &0\\ \vdots &\vdots &\ddots &\vdots \\ 0&0&\cdots \ &{I}_{Ai}\end{array}\right)\left(\begin{array}{l}{\alpha }_{1}\\ {\alpha }_{2}\\ \vdots \\ {\alpha }_{T}\end{array}\right)+\left(\begin{array}{llll}{X}_{1i,0}&{X}_{1i,1}&\ldots &{X}_{1i,K}\\ {X}_{2i,0}&{X}_{2i,1}&\cdots \ &{X}_{2i,K}\\ \vdots &\vdots &\ddots &\vdots \\ {X}_{Ti,0}&{X}_{Ti,1}&\cdots \ &{X}_{Ti,K}\end{array}\right)\left(\begin{array}{l}{\beta }_{t,0}\\ {\beta }_{t,1}\\ \vdots \\ {\beta }_{t,K}\end{array}\right)+\left(\begin{array}{l}{\epsilon }_{1i}\\ {\epsilon }_{2i}\\ \vdots \\ {\epsilon }_{Ti}\end{array}\right)$$

or

5 $${y}_{ti}={{{{\boldsymbol{I}}}}}_{{{{\boldsymbol{Ai}}}}}{{{{\boldsymbol{\alpha }}}}}_{{{{\boldsymbol{t}}}}}+{{{{\boldsymbol{X}}}}}_{ti,k}^{\prime}{{{{\boldsymbol{\beta }}}}}_{{{{\boldsymbol{t}}}},{{{\boldsymbol{k}}}}}+{\epsilon }_{ti}$$

Subscript *t* in Eq. (5) illustrates that the considered outcomes, *y*, and background characteristics, ***X***, can be time-variant. Treatment assignment *I*_*Ai*_ is time-invariant, but parameter *α* can be estimated for each period *t* and *α*_*t*_ represents the ATE in period *t*.

This model can be estimated with a structural estimation model (SEM) or by means of seemingly unrelated regression (SUR), such that it takes into account that the error-terms *ε*_*ti*_ may be correlated within *i*.

### Recurrent individual treatment assignment

In the conventional setting introduced in Section “Results”, individuals are initially randomly assigned to one of both interventions and this assignment status is then maintained throughout the experimental window. The methodological design of this particular setting ensures (1) that an unbiased estimate of the differential ATE in period *t* is obtained, and (2) that it can be evaluated whether this ATE changes—or persists—over time. A baseline approach with respect to treatment assignment based on the derived ATE is to assign future individuals to the on average most effective intervention with *certainty* (i.e., deterministic).

As explained, a major drawback is that heterogeneous treatment effects are not taken into account, yielding sub-optimal individual treatment assignments^5^. In the simple case that all heterogeneity is known, observed, *and* captured by one single individual background characteristic, a moderator effect can be included in the estimation model. For example, if intervention *A* (*B*) is structurally more effective for all women (men), then the inclusion of an intervention interaction term with gender yields an unbiased differential treatment effect for both sub-populations.

However, in practice, it is unclear how the effects of both interventions will vary across individual characteristics^8^. Depending on the relative complexity of the heterogeneity and richness of the data available, some of the variation in individual treatment response may be accounted for, but cannot prevent treatment mistargeting resulting from unobserved dimensions of treatment effect heterogeneity. Formalizing the aforementioned, assume that the effects of interventions *A* and *B* are heterogeneous in observed and unobserved characteristics in the following manner:

6 $${h}_{I}({L}_{i},{X}_{i})={u}_{I}({L}_{i})+{o}_{T}({X}_{i})+{u}_{I}({L}_{i})\cdot {o}_{I}({X}_{i})\quad {{{\rm{for}}}}\,I=A,B.$$

Function *h*_T_( ⋅ ) represents the treatment effect function for intervention *I*, which consists of the functions *u*_I_(*L*_*i*_) and *o*_I_(*X*_*i*_) that represent the variation in treatment effect caused by, subsequently, unobserved (*L*_*i*_) and observed characteristics (*X*_*i*_). The product of *u*_I_(*L*_*i*_) ⋅ *o*_I_(*X*_*i*_) indicates that the interaction of observed and unobserved characteristics may further elicit variation among ITE. Consider the specific case in which treatment effects *A* and *B* vary only with (*one*) observed respondent characteristics (i.e., $$\begin{array}{c}{h}_{I}({L}_{i},{X}_{i})={o}_{I}({X}_{i})\end{array}\ {{{\rm{for}}}}\,I=A,B.$$). For simplicity, and without loss of generality, assume that this characteristic, *X*_1*i*_, is binary. As indicated, such heterogeneity can be accounted for by estimating the baseline model presented in Equation (3), but now with the inclusion of a moderator effect:

7 $${y}_{i}=({\alpha }_{1}+{\alpha }_{2}{X}_{1i})\cdot {I}_{Ai}+{{{{\boldsymbol{X}}}}}_{{{{\boldsymbol{i}}}}}^{\prime}{{{\boldsymbol{\beta }}}}+{\varepsilon }_{i}.$$

Equation (7) shows that the estimated differential treatment effect is (*α*_1_ + *α*_2_*X*_1*i*_) ⋅ *I*_*Ai*_. Based on the estimated differential effectiveness an assignment policy can be formulated that assigns individuals to intervention *A* or *B* conditional on their *X*_1*i*_-status. The effect of having received intervention A (i.e., *I*_*Ai*_ = 1) and not B is

$$\left\{\begin{array}{ll}{\alpha }_{1}\quad &{{{\rm{if}}}}\,{X}_{1i}=0\\ ({\alpha }_{1}+{\alpha }_{2})\quad &{{{\rm{if}}}}\,{X}_{1i}=1\end{array}\right.$$

Let us assume, for expositional purposes, that *α*_1_ = −0.2 and *α*_2_ = 0.6. For persons with characteristic *X*_1*i*_ = 1 the estimation results suggest that the effect of being assigned to intervention *A* and not *B* is (*α*_1_ + *α*_2_) = (−0.2 + 0.6) = 0.4. The assignment policy for persons with characteristic *X*_1*i*_ = 1 would then be to assign them to intervention *A*. In a similar fashion, the assignment policy for persons with characteristic *X*_1*i*_ = 0 is to assign them to intervention *B* because the effect of being assigned to intervention *A* and not *B* is −0.2.

However, if the treatment effect varies with unobserved characteristics, then the inclusion of a moderator effect is not sufficient to account for this unobserved heterogeneity. Also, more advanced modeling approaches towards estimating *HTE*—or even *ITE*—cannot address unobserved dimensions of heterogeneity.

It is because of this fundamental problem of modeling unobserved heterogeneity in treatment effects, that the Recurrent Individual Treatment Assignment (*RITA*) approach presented below refrains from attempting to estimate ITE and focuses on individual treatment response and assignment instead. *RITA* learns (iteratively) over time how treatment response development varies across—and within—individuals and updates assignment decisions accordingly, without explicitly modeling treatment effect heterogeneity. Since variation in individual treatment response encompasses all heterogeneity, *RITA* has the potential to accommodate both observed and unobserved heterogeneous treatment effects.

### A RITA algorithm

We first outline the formal steps for recurrent individual treatment assignment (*RITA*), such that observed and unobserved heterogeneity can be detected and accounted for in making sequential treatment assignment decisions. Then, we intuitively explain the rationale behind each step of such a *RITA* algorithm.

Let *y*_0*i*_ be the (baseline) outcome observed before initial treatment assignment and *obs* be the number of individuals for which iterative assignment has to be determined. In each period *t*, *RITA* then executes the following sequence of steps:

1. Assign individual *i* randomly to intervention *A* with probability *P*_*ti*_(*A*) = *τ*_*Ati*_ and to intervention *B* with probability *τ*_*Bti*_ = (1−*τ*_*Ati*_) in period *t*.
2. Determine treatment response for individual *i* in period *t* (Δ*y*_*t*,*i*_ = *y*_*t*,*i*_−*y*_*t*−1,*i*_).
3. Determine the rank in distribution Δ*y*_*t*,*i*_ for individual *i* in period *t*, (rank_*ti*_(Δ*y*_*t*,*i*_)).
4. Individual mean rank (IMR) for intervention *A* (IMR_*Ati*_), conditional on the cumulative frequency of being assigned *A* :
  8 $${\mathrm {IM{R}}}_{Ati}=\left\{\begin{array}{ll}\frac{\mathop{\sum }\limits_{[t=1]}^{T}{\mathrm {ran{k}}}_{Ati}({{\Delta }}{y}_{t,i})}{\mathop{\sum }\limits_{[t=1]}^{T}{c}_{Ati}}\quad &{{{\rm{if}}}}\,{I}_{t}=1\\ \hspace{1em}{\mathrm {IM{R}}}_{At-1,i}\quad &{{{\rm{if}}}}\,{I}_{t}=0\end{array}\right.$$
  and similarly, update IMR for intervention *B* (IMR_*Bti*_):
  9 $${\mathrm {IM{R}}}_{Bti}=\left\{\begin{array}{ll}\frac{\mathop{\sum }\limits_{[t=1]}^{T}{\mathrm {ran{k}}}_{Bti}({{\Delta }}{y}_{t,i})}{\mathop{\sum }\limits_{[t=1]}^{T}{c}_{Bti}}\quad &{{{\rm{if}}}}\,| ({I}_{t}-1)| =1\\ \hspace{1em}{\mathrm {IM{R}}}_{Bt-1,i}\quad &{{{\rm{if}}}}\,| ({I}_{t}-1)| =0\end{array}\right.$$
5. Update treatment assignment probability *P*_*t*+1,*i*_(⋅) for individual *i* in period *t* + 1:
  10 $${P}_{t+1,i}(\cdot )=\left\{\begin{array}{ll}\left\{\begin{array}{l}{\tau }_{At+1,i}=[\frac{{\mathrm {ran{k}}}_{Ati}({{\Delta }}{y}_{t,i})}{{\mathrm {obs}}}]\cdot {[\frac{{\mathrm {ran{k}}}_{Ati}({{\Delta }}{y}_{t,i})}{\mathrm {{IM{R}}}_{Bti}}]}^{(\mathop{\sum }\limits_{[t = 1]}^{T}{c}_{Ati}-1)}\quad \\ 1-{\tau }_{At+1,i}\quad \end{array}\right.\quad &{{{\rm{if}}}}\,{I}_{ti}=1\\ \left\{\begin{array}{l}{\tau }_{At+1,i}\quad \\ 1-{\tau }_{At+1,i}=[1-\frac{{\mathrm {ran{k}}}_{Bti}({{\Delta }}{y}_{t,i})}{{\mathrm {obs}}}]\cdot {[\frac{{\mathrm {IM{R}}}_{Ati}}{\mathrm {{ran{k}}}_{Bti}({{\Delta }}{y}_{t,i})}]}^{(\mathop{\sum }\limits_{[t = 1]}^{T}{c}_{Bti}-1)}\quad \end{array}\right.\quad &{{{\rm{if}}}}\,{I}_{ti}=0\end{array}\right.$$
6. Bound *P*_*t*+1,*i*_(⋅) to ensure that RITA maintains a minimally desired level of exploration:
  10 $${P}_{t+1,i}(Z)=\left\{\begin{array}{ll}{\tau }_{Zt+1,i}={\lambda }_{u}\quad &{{{\rm{if}}}}\,{\tau }_{Zt+1,i}\ge {\lambda }_{u}\\ {\tau }_{Zt+1,i}={\lambda }_{l}\quad &{{{\rm{if}}}}\,{\tau }_{Zt+1,i}\le \,{\lambda }_{l}\end{array}\right.\qquad {{{\rm{for}}}}\,Z=A,B$$

For expositional purposes, assume that the above *RITA algorithm* is applied on a sample of 1000 participants (i.e., *obs* = 1000). In the first period (*t* = 1), no evidence has yet emerged to distinguish between intervention *A* and *B*. Therefore the initializing values of the individual mean ranks are IMR_*A*0*i*_ = IMR_*B*0*i*_ = $\frac{1}{2}.{\mathrm {obs}}=500,$ such that each individual is randomly assigned to intervention *A* or *B* with probability 0.5 (i.e., ${\tau }_{A1i}={\tau }_{B1i}=\frac{1}{2}$) in Step 1. Step 2 determines treatment response for every individual *i* by observing the difference in outcome scores between periods 1 and 0.

In Step 3 each individual receives a ranking that is based on the outcome change observed (i.e., based on intervention response) and conditionally on the intervention received. With 1000 participants, the assigned rank-value is 1 for the smallest outcome-change and 1000 for the largest (i.e., most beneficial) outcome-change. This rank function thus determines for whom the outcome improvement was greatest. We note that the observed intervention responses (i.e., rank_*ti*_(Δ*y*_*t*,*i*_)) contain individuals who received intervention A and who received intervention B. Suppose that an individual was assigned to intervention A and experiences a small outcome-change, then the algorithm will more likely assign this person to the other intervention. However, also when receiving the other intervention, this person may end up low in the outcome-change distribution. Intuitively, the algorithm thus iteratively determines the relative effectiveness of both interventions at the individual level, using intervention response and not by estimating the treatment effect.

Step 4 updates the individual mean rank (IMR) for either intervention *A* or *B*. To illustrate this step, consider a participant Dina who was assigned to intervention *A* in period 1, and that the rank associated with her outcome-gain is 600. From Eqs. (8) and (9) it follows that her individual mean rank is updated to 600 for intervention *A* and (remains) 500 for intervention *B*. Note that ∑*c*_*Ati*_ (${\sum }_{[t=1]}^{T}{c}_{Bti}$) indicates the cumulative frequency of being assigned to intervention *A* (*B*) and the individual mean rank is only updated for the intervention that individual *i* was assigned to in period *t*.

These individual mean ranks are then used in Step 5 to update the individual treatment assignment probabilities for period *t* + 1, using all the information observed in the previous *t* periods. Equation (10) updates this probability conditional on (1) the rank-values observed in period *t* and (2) on the intervention received. In the example of Dina, who was assigned intervention *A* in period *t* = 1, the updating rule for intervention condition *I*_*ti*_ = 1 thus applies. To grasp the intuition of this treatment assignment rule, consider its 3 separate components:

$$\begin{array}{l}[\frac{{\mathrm {ran{k}}}_{Ati}({{\Delta }}{y}_{t,i})}{{\mathrm {obs}}}]\cdot {[\frac{{\mathrm {ran{k}}}_{Ati}({{\Delta }}{y}_{t,i})}{{\mathrm {meanran{k}}}_{Bti}({{\Delta }}{y}_{t,i})}]}^{(\mathop{\sum }\limits_{[t = 1]}^{T}{c}_{Ati}-1)}\\ =\\ {[{\mathrm {Individual}}\,{\mathrm {Rank}}]\cdot [{\mathrm {Relative}}\,{\mathrm {Rank}}]}^{({\mathrm {Learning}}\,{\mathrm {Parameter}})}\end{array}$$

The first component between brackets represents the re-scaled individual rank in the rank distribution, which for Dina is thus 0.6 (i.e., $\frac{600}{1000}=0.6$). The second component between brackets represents the relative rank and evaluates whether the observed rank in period *t* is higher or lower than the mean rank of the alternative intervention. For Dina, the mean rank of the alternative intervention (to which she has never been assigned to after period *t* = 1) equals $\frac{1}{2}\cdot {\mathrm {obs}}=500$, such that the relative rank is $\frac{600}{500}=1.2$. The value of the relative rank is thus higher than 1 if intervention A appears relatively more effective after period *t* and between 0 and 1 if the opposite holds.

The third component can be considered a learning parameter that is set to 0 in period 1, such that the treatment assignment probability is updated by the individual rank only. With each period that an individual is assigned to intervention *A* or *B*, the algorithm has learned more about whether that is the best intervention available. This is reflected by the corresponding learning parameter increase with 1, such that the importance of the relative rank in updating the treatment assignment probability increases exponentially. This is a critical feature of the *RITA*-algorithm in that it pushes individuals towards the most effective intervention as more instances of treatment response are observed. At the same time, the treatment effect may change dynamically over time, such that the ‘best’ intervention changes from A to B or vice-versa. If this happens, the exponential influence of the relative rank ensures that *RITA* can learn quickly and swiftly reallocates individuals to a different intervention instead.

To illustrate the exponential nature of this learning parameter, consider again Dina. Her treatment assignment probability for intervention *A* in period 2 (*τ*_*A*1+1,*i*_) equals 0.6 ⋅ (1.2)^0^ = 0.6. Suppose that Dina is again assigned to intervention *A* in period 2 and that her individual rank in this period is again 0.6, such that her relative rank maintains a value of 1.2. Her treatment assignment probability for intervention *A* in period 3 (*τ*_*A*2+1,*i*_) then increases to 0.6 ⋅ (1.2)^1^ = 0.72. The learning parameter thus operates as a drift factor, such that the relative rank importance is increasing in the number of observations that it is based on.

The final Step 6 addresses two remaining issues. Firstly, the generated treatment assignment probabilities in Step 5 can exceed 1. Secondly, ITE maybe not be stable over time (i.e., maybe dynamic). In order to acknowledge the potential for such within-individual variation, it might be desirable to maintain a minimum pre-specified level of exploration at the expense of fully exploiting the information from previous observations (i.e., explore-exploit trade-off). To allow for exploration to be maintained, upper and lower probability bounds (*λ*_*u*_ and *λ*_*l*_) can be imposed in *RITA*. In this study, these minimum exploration bounds are set to *λ*_*u*_ = 0.95 and *λ*_*l*_ = 0.05, such that treatment assignment will never be fully deterministic. It follows that maintaining exploration by will lead to a sub-optimal performance in a setting in which individual dynamic treatment effects turn out not to exist, which is inherent to the explore-exploit trade-off.

Step 6 of the algorithm intuitively states that, even when the previous 5 steps indicate that a person should be assigned to intervention with certainty, the algorithm must continue to explore to a certain extent. In this study, the exploration parameter is set at 0.05. Given that intervention, effects may be dynamic, and given the discussion with respect to the drift parameter, exploration is important, as it occasionally assigns participants to the supposedly suboptimal treatment to check whether this nonoptimality confirmed. If the upcoming iteration (and due to dynamic changes in intervention effects) for the supposedly non-optimal treatment assignment gives that participant a high ranking, then the drift parameter ensures that the algorithm can quickly react on that. On the other hand, if in the upcoming iteration the non-optimal treatment assignment is confirmed, then the algorithm will again set the exploration parameter at 0.05 and assign the participant to the best performing treatment with 0.95 percent certainty.

### RITA and accounting for heterogeneity

The key advantage of RITA over other HTE-modeling approaches based on observable characteristics (i.e., conditional frequentist and stochastic approaches) is that it focuses on intervention response. By recognizing that all heterogeneity is encompassed by variation in intervention response. RITA accounts for observed and unobserved heterogeneity (i.e., *h*_*I*_(*L*_*i*_, *X*_*i*_)) without explicitizing the nature of the heterogeneity (in advance). As such, both observed and unobserved heterogeneity is acknowledged in determining treatment assignment. This is the result of combining the RCT setting with insights from predictive modeling.

In addition to improving individual treatment assignment, *RITA* can indicate how optimal treatment assignments are structurally related to observed background characteristics (i.e., *o*_T_(*X*_*i*_)). Therefore, *RITA* can reveal that, for example, one treatment is assigned to a particular subgroup, while another treatment is assigned to another particular subgroup. An examination of the characteristics of these subgroups can reveal important information concerning *why* a particular intervention is effective (the diagnosis). *RITA* thus gives insights into for which baseline characteristics a particular treatment is effective, yielding the potential to reveal the underlying treatment effect mechanisms. We note that the interpretation itself of this structural relationship, which translates into the diagnosis, is not a task for *RITA*, but more a task for field experts. But, acknowledging that *RIT*A can reveal such structural relationships also implies that the environment that implements *RITA* should try to collect information potentially related to the underlying mechanisms as to know why the interventions can be effective.

### Reporting summary

Further information on experimental design is available in the Nature Research Reporting Summary linked to this article.

## Supplementary information

Reporting Summary

## Data Availability

The simulated data of the current study are available from the corresponding author on request.
